# Prescribing of nicotine replacement therapy in and around pregnancy: a population-based study using primary care data

**DOI:** 10.3399/bjgp14X681361

**Published:** 2014-09-01

**Authors:** Nafeesa N Dhalwani, Lisa Szatkowski, Tim Coleman, Linda Fiaschi, Laila J Tata

**Affiliations:** Division of Primary Care, and doctoral student, Division of Epidemiology and Public Health, University Of Nottingham, Nottingham, UK.; Division of Epidemiology and Public Health, University Of Nottingham, Nottingham, UK.; Division of Primary Care, University Of Nottingham, Nottingham, UK.; Division of Epidemiology and Public Health, University Of Nottingham, Nottingham, UK.; Division of Epidemiology and Public Health, University Of Nottingham, Nottingham, UK.

**Keywords:** nicotine replacement therapy, pregnancy, prescribing, smoking cessation

## Abstract

**Background:**

Licensing arrangements for nicotine replacement therapy (NRT) in the UK were broadened in 2005 to allow prescribing to pregnant smokers. However, estimates of NRT prescribing in pregnant females in the UK are currently lacking.

**Aim:**

To assess trends in NRT prescribing around pregnancy, and variation in prescribing by maternal characteristics.

**Design and setting:**

Population-based descriptive study using pregnancy data from The Health Improvement Network primary care database, 2001–2012.

**Method:**

NRT prescriptions were identified during pregnancy and in the 9 months before and after. Annual prescribing prevalence was calculated. Logistic regression was used to assess females’ likelihood of receiving prescriptions by maternal characteristics.

**Results:**

Of 388 142 pregnancies studied, NRT was prescribed in 7551 for an average duration of 2 weeks. The prescribing prevalence of NRT increased from 0.03% (0.7% in smokers) in 2001 to 2.6% (11.4% in smokers) in 2005, after which it remained stable. Prescribing prevalence of NRT before and after pregnancy was half the prevalence during pregnancy. The odds of prescribing NRT during pregnancy in smokers increased with socioeconomic deprivation (OR = 1.29, 95% CI = 1.15 to 1.45 in the most compared with the least deprived group). Prescribing was 33% higher in pregnant smokers with asthma (OR = 1.33, 95% CI = 1.22 to 1.45) and mental illness (OR = 1.33, 95% CI = 1.23 to 1.44) compared with smokers without these diagnoses.

**Conclusion:**

NRT prescribing is higher during pregnancy compared with before and after, and is higher in smokers from more socioeconomically deprived groups, those with asthma or those diagnosed mental illness.

## INTRODUCTION

Smoking in pregnancy is related to several adverse outcomes for both mothers and their children.^1^,^2^ In the UK, 26% of mothers smoke directly before or during their pregnancy, and 12% continue to smoke throughout.^3^ Similar prevalence has been reported in Australia and the US (11.7% and 10.7% respectively).^4^,^5^ Therefore, reducing smoking in pregnancy is a global public health priority.^6^

Nicotine replacement therapy (NRT) is a pharmacological smoking cessation aid which became available on prescription from the UK NHS in April 2001.^7^ It was initially contraindicated during pregnancy because of a lack of evidence for its safety.^8^ To date there is no conclusive evidence on its effectiveness during pregnancy,^9^ and studies of NRT safety during pregnancy are inconclusive.^2^,^10^–^12^ Nevertheless, expert consensus is that NRT is likely to be less harmful than smoking during pregnancy and, with various caveats, NRT has been recommended by international guidelines when smoking cessation without NRT is unsuccessful.^13^–^16^

Literature describing NRT use in pregnancy is limited to observational studies from the US and Denmark assessing the association of NRT use during pregnancy and adverse birth outcomes. The prevalence of self-reported NRT used in the first 12 weeks of gestation was 0.3%,^17^ 2–2.5% in 17–27 weeks of gestation in the Danish National Birth Cohort,^10^,^11^ and in the Pregnancy Risk Assessment Monitoring System (PRAMS) from four US states it was 3.9% (2004).^2^ Since their publication, new NRT products have been introduced and international guidelines on gestational NRT use have changed. In 2013, the World Health Organization (WHO) recommended an urgent need for studies on the surveillance of current NRT use in pregnancy.^16^

In December 2005, UK licensing arrangements were changed to allow prescribing of NRT to pregnant smokers.^18^ As a result, NRT can now be prescribed to pregnant females by GPs, midwives, or other licensed health professionals working in NHS Stop Smoking Services (SSS) after discussing the risks and benefits of using the drug in pregnancy. Although it can also be bought directly from pharmacies or other retailers such as supermarkets, all drug packaging retains warnings against its use in pregnancy without prior GP consultation. Most NRT is probably received via GP prescription, as half of NRT provided by pregnancy SSSs is issued via the patient’s GP.^19^

Thus far, only two UK studies have assessed NRT use in pregnancy.^20^,^21^ One of these studies only presents local data from Tayside, Scotland,^21^ and the second was only among females attending NHS SSS in England.^20^ Given that only 3% of all pregnant females attend SSSs, this will have excluded most pregnant smokers.^22^,^23^ In this study, UK prescribing of NRT is quantified before, during, and after pregnancy using a nationally representative sample, and the characteristics of females who receive NRT prescriptions are investigated.

**How this fits in**

Pregnancy is an opportunistic time to offer smoking cessation interventions to females. This study is the first to quantify prescribing of nicotine replacement therapy before, during, and after pregnancy in the UK. Prescribing prevalence of nicotine replacement therapy during pregnancy was 11% among smokers, double the prescribing prevalence before and after pregnancy. However, most females received only 2 weeks of nicotine replacement therapy during pregnancy. Prescribing was higher in pregnant smokers from more deprived areas and in smokers with diagnoses of asthma or mental illness.

## METHOD

### Data source and study population

The Health Improvement Network (THIN), an electronic database containing anonymised patient records from general practices across the UK, was used for this study, covering approximately 6% of the population,^24^ representative of the UK population in terms of demographics, prevalence of common illnesses, and fertility rates.^25^,^26^ Prevalence of smoking and prescribing of smoking cessation medications in the general population in THIN has been validated against national data.^27^,^28^ The study population included all pregnancies between January 2001 and December 2012 in females of childbearing age (15–49 years), resulting in a live birth or a stillbirth.

### Outcome and covariates

The smoking status of females was determined using Read Codes^29^ recorded from 27 months before conception up to the end of pregnancy, based on the recording rules in the GP contract,^30^ which is described in detail elsewhere.^31^ Multilex Drug Codes for all NRT formulations available in the UK according to the *British National Formulary* (*BNF*) were used for NRT prescriptions.^32^ Code lists are available from the authors on request.

To investigate factors associated with NRT prescribing, data were extracted on females’ age at conception, socioeconomic deprivation (Townsend Index),^33^ preconception body mass index (BMI), and diagnoses of medical conditions (hypertension, diabetes, asthma, and mental illness, which included depression, anxiety, bipolar disorder, schizophrenia, and other psychoses) during or before pregnancy. These conditions were selected as they are closely related to smoking,^3^,^34^–^38^ and may influence quit attempts.

### Statistical analysis

Overall and annual proportions of pregnancies, and pregnancies among smokers, with one or more NRT prescriptions before, during and after pregnancy were determined. There is no evidence of the time before and after pregnancy during which smokers are more likely to attempt to quit, therefore the 9 months before and after pregnancy were used to calculate prescribing prevalence, as these were similar to the average pregnancy length, allowing for comparisons of period prevalence. As smoking behaviours may fluctuate during a 9-month period, 3-month windows were also assessed during and around pregnancy. The use of different forms of NRT (patches, gum, nasal spray, lozenges, sublingual tablets, inhalator cartridges, and combination) was assessed.

Logistic regression was used to calculate ORs for associations between females’ characteristics and prescribing of NRT to smokers during pregnancy, restricting to pregnancies delivered from January 2006, after relaxation of licensing arrangements.^18^ All covariates reaching statistical significance at the 5% level in univariable models were included in the multivariable analysis and each covariate was sequentially dropped from the model to assess whether it remained statistically significant, retaining only those that were. Some females had more than one pregnancy during the study period and there may be potential clustering of females within practices; this was accounted for by using generalised estimating equations (GEE) with an exchangeable correction structure which provided best estimates of the population-level associations with maternal characteristics despite potential dependence between pregnancy, that is accounting for clustered data.^39^ Analyses were performed using Stata (version 12.0).

## RESULTS

### Baseline characteristics

Between 2001 and 2012, 388 142 pregnancies were identified resulting in live births or stillbirths, of which 71 685 (18.5%) were in smokers. Mean age at conception was 29.6 years (SD 5.9). Table 1 describes females’ characteristics for all pregnancies and pregnancies among smokers, and NRT prescribing according to these characteristics.

**Table 1.** Baseline characteristics of the study population

	**Total pregnancies, *n* = 388 142**	**NRT prescribed, total *n* = 7551 (% of pregnancies with NRT prescription)**	**Pregnancies among smokers,^a^*n* = 71 685**	**NRT prescribed, *n* = 7551 (% of pregnancies among smokers with NRT prescription)**
**Age at conception, years**				
5–19	27 365	930 (3.4)	9898	930 (9.4)
20–24	66 484	1962 (3.0)	19 849	1962 (9.9)
25–29	105 967	2004 (1.9)	19 188	2004 (10.4)
30–34	118 031	1664 (1.4)	14 534	1664 (11.4)
35–39	59 541	832 (1.4)	6952	832 (12.0)
40–44	10 222	152 (1.5)	1203	152 (12.6)
45–49	532	7 (1.3)	61	7 (11.5)

**Townsend score in quintiles^b^**				
Quintile 1 – most affluent	83 203	722 (0.9)	7970	722 (9.1)
Quintile 2	71 045	925 (1.3)	9511	925 (9.7)
Quintile 3	76 619	1432 (1.9)	14 291	1432 (10.0)
Quintile 4	73 470	2068 (2.8)	18 097	2068 (11.4)
Quintile 5 – most deprived	55 653	1875 (3.4)	16 925	1875 (11.1)
Missing	28 152	529 (1.9)	4891	529 (10.8)

**Pre-conception body mass index, kg/m^2^**				
Normal (18.0–24.9)	118 832	2266 (1.9)	22 888	2266 (9.9)
Underweight (<18.0)	8614	256 (3.0)	2353	256 (10.9)
Overweight (25–29.9)	58 693	1237 (2.1)	11 464	1237 (10.8)
Obese (≥30)	42 281	992 (2.3)	9191	992 (10.8)
Missing	159 722	2800 (1.8)	25 789	2800 (10.9)

**Asthma**	33 724	1061 (3.1)	8188	1061 (13.0)

**Hypertension**	9992	154 (1.5)	1420	154 (10.8)

**Diabetes**	10 752	224 (2.1)	1798	224 (12.5)

**Mental illness**	37 055	1547 (4.2)	11 624	1547 (13.3)

a Smoker classified as those with a record of current smoking at some point within the 27 months before conception until delivery.

b Socioeconomic status. NRT = nicotine replacement therapy.

### Patterns of NRT prescribing in and around pregnancy

**Figure 1.**
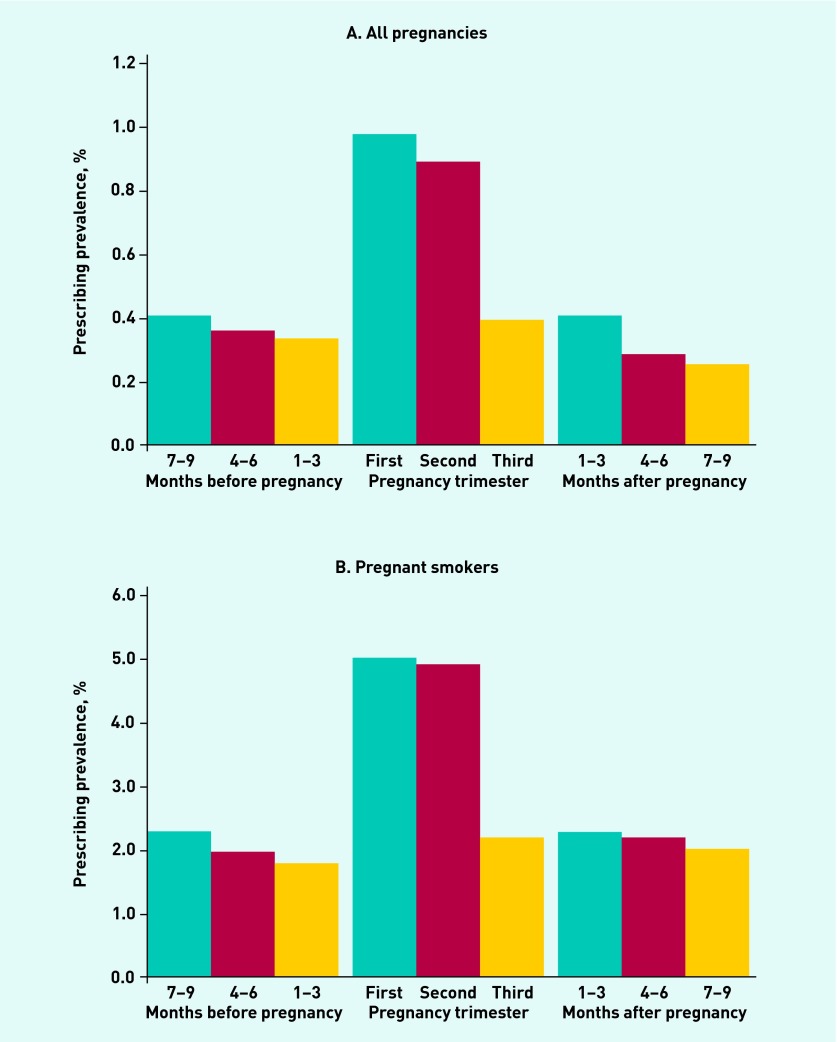
***Proportion of overall pregnancies and smokers with NRT prescriptions in each 3-month time period, between 2001 and 2012.***

NRT was prescribed in 7551 pregnancies, which represented a prescribing prevalence of 2% of all pregnancies and 11% of pregnancies in smokers. In comparison, the prescribing prevalence was 1% during the 9 months before and after pregnancy overall, and 5% in smokers. Figure 1 shows the prescribing prevalence in 3-month periods outside pregnancy and by trimester. NRT prescribing among smokers was most frequent during the first and second trimesters at just over 5%, compared with 2% in the third trimester.

Among the pregnancies where NRT was prescribed, over half (55%) had only one prescription issued, 25% had two prescriptions, and 20% had three or more prescriptions. The maximum number of prescriptions issued during pregnancy was 26. On average, females were prescribed a total of 2 weeks’ worth of NRT (interquartile range 1–2 weeks). The prescription frequency and length of NRT issued in the 9 months before and after pregnancy was similar to pregnancy time.

In two-thirds of the pregnancies in which NRT was prescribed, it was initiated only during pregnancy, with no evidence of NRT prescribing prior to the start of pregnancy. The most common form of NRT used during pregnancy was transdermal patches (65% of all prescriptions), followed by inhalator cartridges (17%), gum (8%), lozenges (6%), sublingual tablets (2%), oromucosal spray (0.7%), and nasal spray (0.3%). Combination NRT was used in 14% of pregnancies where NRT was prescribed. The distribution of NRT forms prescribed before and after pregnancy was very similar.

### Annual prescribing of NRT before, during, and after pregnancy

**Figure 2.**
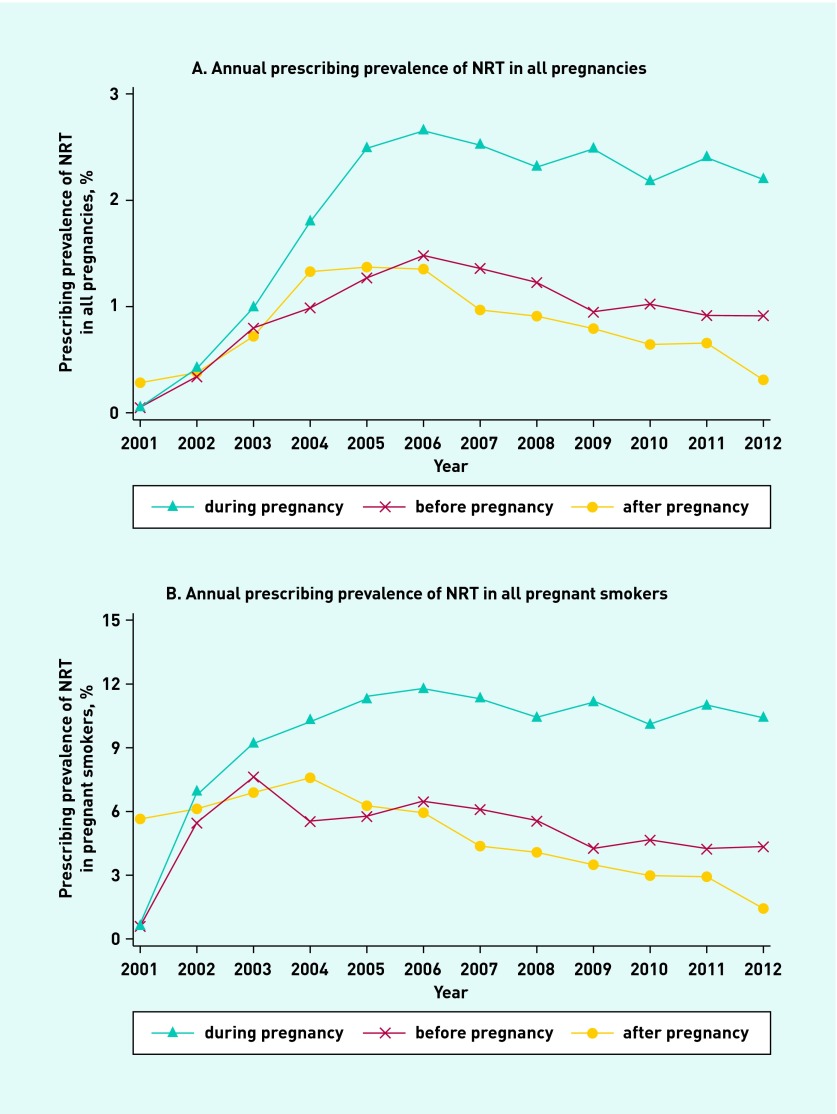
***Annual prescribing prevalence of NRT between 2001 and 2012.***

Figure 2 shows the proportion of pregnancies between 2001 and 2012 in which NRT was prescribed before, during, and after pregnancy. In 2001, the prescribing prevalence of NRT in all pregnancies during gestation was 0.03% (0.7% of pregnancies among smokers). This increased to 2.6% (11.4% among smokers) in 2005, after which it remained stable. The proportion of pregnancies with NRT prescriptions issued in the 9 months before and after pregnancy increased until 2004, after which it remained stable at around 1% (6% among smokers) with a gradual decline in prescribing prevalence after 2006.

### Prescribing of NRT by maternal characteristics

Table 2 shows the maternal characteristics associated with prescribing in pregnant smokers between 2006 and 2012. Prescribing was higher in older compared with younger age groups (OR for 40–44 years = 1.27, 95% CI = 1.03 to 1.58, compared with the 25–29-year age group). Pregnant smokers from more socioeconomically deprived groups were more likely to receive prescriptions compared with less deprived groups (OR for quintile 5 compared with quintile 1 = 1.29, 95% CI = 1.15 to 1.45). Pregnant smokers with a diagnosis of asthma or mental illness were 33% more likely to be prescribed compared with pregnant smokers without these morbidities.

**Table 2.** Prescribing of NRT in pregnant smokers by maternal characteristics between January 2006 and December 2012

**Demographic variables**	**Pregnancies among smokers with one or more NRT prescriptions, *n* = 5756,^a^*n* (%)**	**Unadjusted odds ratio (95% CI)**	***P*- value**	**Adjusted odds ratio (95% CI)^b^**	***P*- value**
**Age at conception, years**					
15–19	721 (9.9)	0.92 (0.84 to 1.01)		0.93 (0.84 to 1.03)	
20–24	1533 (10.2)	0.94 (0.88 to 1.02)	<0.001	0.93 (0.86 to 1.00)	<0.001
25–29	1537 (10.7)	1.00		1.00	
30–34	1222 (11.9)	1.12 (1.04 to 1.22)		1.13 (1.04 to 1.23)	
35–39	616 (12.2)	1.16 (1.05 to 1.28)		1.17 (1.05 to 1.30)	
40–44	122 (13.3)	1.28 (1.05 to 1.56)		1.27 (1.03 to 1.58)	
45–49	5 (10.9)	1.01 (0.40 to 2.58)		1.12 (0.43 to 2.96)	

**Townsend score**					
Quintile 1 (least deprived)	505 (9.2)	1.00		1.00	
Quintile 2	696 (10.1)	1.10 (0.98 to 1.25)	<0.001^c^	1.09 (0.96 to 1.24)	<0.001^c^
Quintile 3	1101 (10.3)	1.14 (1.01 to 1.27)		1.19 (1.05 to 1.34)	
Quintile 4	1605 (11.8)	1.35 (1.19 to 1.48)		1.37 (1.22 to 1.54)	
Quintile 5 (most deprived)	1428 (11.3)	1.25 (1.13 to 1.39)		1.29 (1.15 to 1.45)	
Missing	421 (11.4)	1.27 (1.10 to 1.45)		1.33 (1.15 to 1.54)	

**Pre-conception body mass index, kg/m^2^**					
Underweight	206 (11.0)	1.12 (0.96 to 1.30)			
Normal	1786 (10.6)	1.00	0.414	–	–
Overweight	976 (11.2)	1.06 (0.97 to 1.14)			
Obese	806 (11.1)	1.06 (0.96 to 1.15)			
Missing	1982 (10.8)	1.02 (0.95 to 1.09)			

**Diabetes**	193 (13.2)	1.26 (1.08 to 1.47)	0.075	–	–

**Hypertension**	114 (11.3)	1.04 (0.86 to 1.27)	0.660	–	–

**Asthma**	845 (13.8)	1.36 (1.26 to 1.48)	<0.001	1.33 (1.22 to 1.45)	<0.001

**Mental illness**	1181 (13.7)	1.38 (1.29 to 1.48)	<0.001	1.33 (1.23 to 1.44)	<0.001

NRT = nicotine replacement therapy.

a Percentages of pregnancies in smokers with NRT prescription.

b All covariates mutually adjusted.

c P*-value for trend.*

## DISCUSSION

### Summary

After NRT was made available on NHS prescription in 2001, prescribing in and around pregnancy increased; by 2005 prescribing was twice as high during pregnancy as in the 9 months immediately before and after pregnancy, despite being contraindicated for pregnant females. The December 2005 licence relaxation to allow prescribing in pregnancy did not further increase these trends and the prescribing prevalence during pregnancy has remained stable at 2% (11% in smokers). Females with asthma or mental illnesses and those from more socioeconomically-deprived areas were more likely to receive prescriptions during pregnancy. Eighty per cent of females received ≤2 prescriptions.

### Strengths and limitations

Using a large population-based data source, longitudinal and contemporaneous prescribing estimates are presented; this is the first study of NRT prescribing during pregnancy in the UK and the only study internationally that has assessed prescribing trends. Ascertainment of NRT use is based on prescribing data rather than self-reported NRT use, which females may under-report.^11^ Prescribing in 9-month periods immediately before and after pregnancy was also assessed, whereas other studies only report NRT use in trimesters one and two.^10^,^11^,^17^ Therefore, the present estimates of NRT prescribing around pregnancy are novel in providing population-level information on smoking cessation attempts pre-conception and postpartum for the first time.

The present study data capture all NRT prescribing to pregnant females in UK primary care in practices registered in THIN. These data may not include NRT prescribing in other settings such as local NHS Stop Smoking Services for Pregnant females (SSSP) and NRT purchased in pharmacies or retailers. A survey of all SSSPs in England conducted between April 2010 and March 2011 reported that almost half of the NRT provided by these services was issued through GPs.^40^ In terms of self-purchased NRT, the authors believe this will be infrequent for several reasons. Firstly, the prevalence of medication use without health professional consultation is lower during pregnancy than when females are not pregnant.^41^ Furthermore, all packages of NRT clearly instruct females to consult a doctor before using them if they are pregnant. Lastly, in the UK females are entitled to free NHS prescriptions during pregnancy,^42^ so they are more likely to get free prescriptions through GPs than paying for NRT. Hence, the authors believe that this study captures most prescriptions of NRT issued and provides valuable information on prescribing patterns during pregnancy.

Potential changes in smoking habits were not accounted for over the study, and therefore this study has also presented proportions for all pregnancies. Some females may quit or relapse after delivery consequently leading to changes in the baseline smokers; NRT estimates could therefore be overestimated if more females relapse than are recorded, and underestimated if more females quit. In the present data, however, over 75% of pregnant females who were classified as smokers during pregnancy and who had a recording of smoking status within the 9 months after delivery were still recorded as smokers. Therefore, a substantial overestimation or underestimation is unlikely.

### Comparison with existing literature

The present study data suggest that UK prescribing of NRT during pregnancy increased between 2001 and 2005, after which it plateaued. Despite NRT use being recommended in smoking cessation guidelines for pregnant females in several countries,^14^,^15^,^18^ no other studies thus far have assessed the annual prescribing prevalence of NRT during pregnancy for comparison. Studies from the US, Denmark, and Scotland report an overall prescribing prevalence of between 0.3% and 4%.^2^,^10^,^11^,^21^ NRT use in pregnant smokers attending English SSSs is reported to be 85%^20^ and considering that only 3% of pregnant females attend these services, this equates to 2.5%, which is similar to the present findings.

The National Institute for Health and Care Excellence (NICE) recommends that pregnant females should initially be prescribed 2 weeks of NRT from their agreed stop date with further NRT after re-assessment.^7^ The average duration of prescription for females in the present study was 2 weeks and most females (80%) received two or fewer prescriptions. One reason for this may be that compliance was low and females did not quit or use it to quit, in which case no further NRT was prescribed. Some females may have bought NRT independently after the first prescription; however, considering that females are entitled to free prescriptions during pregnancy and NRT from retailers is reasonably expensive, this is unlikely. Studies in other populations have not reported the duration of NRT use in pregnancy. However, 8–12 weeks’ use is recommended for optimal effectiveness in the general population,^43^ so it is unlikely that 2 weeks’ use is effective for smoking cessation in pregnancy.

A study including 5716 pregnant females from the US showed NRT prescribing to be lower in pregnant smokers aged <35 years compared with pregnant smokers aged ≥35 years,^2^ which is similar to the present findings. Low socioeconomic status is associated with a higher prevalence of chronic disease^44^ and higher risk of adverse pregnancy outcomes,^45^ which could explain why pregnant smokers in the deprived group in this study were prescribed NRT more often than affluent groups. Asthma and mental illness are the most common medical conditions encountered during pregnancy,^46^,^47^ and are closely related to smoking, which may explain the significant association with NRT prescribing compared with other conditions.

The English SSSPs study reported that 55% of all pregnant smokers (65% of pregnant NRT users) used combination NRT,^20^ which is high compared with the present estimate of 14%. This is mostly likely related to different baseline populations. Females voluntarily attending these specialist services likely have a higher motivation to quit, which may result in more quit attempts and more NRT being prescribed compared with females attending primary care.

It is unfortunate that NRT prescribing prevalence outside pregnancy began to decline considering the demonstrated effectiveness of NRT;^48^ however, this may be related to the licensing of varenicline for smoking cessation in the non-pregnant population in December 2006, after which a reduction in NRT and bupropion prescribing was seen in the general population.^49^

### Implications for practice

Pregnancy offers a strategic opportunity for health professionals to promote smoking cessation as females are generally more receptive to cessation interventions and are more likely to attempt to quit smoking because of the potential foetal harm associated with smoking during pregnancy.^50^ The study findings give insight into the prescribing in and around pregnancy, which is important for policy makers and GPs to monitor and promote smoking cessation in females of childbearing age. The study shows that NRT was prescribed for an average of only 2 weeks during pregnancy, which is unlikely to be effective considering that NRT use in the general population for smoking cessation is recommended for at least 8–12 weeks. It is also highlighted that only 1% of smokers who are not yet pregnant receive NRT, which indicates missed opportunities to assist young females to quit, despite the reported effectiveness of NRT outside pregnancy. Although interactions between health professionals and females during pregnancy should be used to discuss and offer interventions to promote smoking cessation, greater potential benefit would result from starting before pregnancy which should be a prioritised focus for females and healthcare providers.
